# A PDGFRβ-PI3K signaling axis mediates periosteal cell activation during fracture healing

**DOI:** 10.1371/journal.pone.0223846

**Published:** 2019-10-30

**Authors:** Laura Doherty, Jungeun Yu, Xi Wang, Kurt D. Hankenson, Ivo Kalajzic, Archana Sanjay

**Affiliations:** 1 Department of Orthopaedic Surgery, UConn Health, Farmington, Connecticut, United States of America; 2 Department of Reconstructive Sciences, UConn Health, Farmington, Connecticut, United States of America; 3 Department of Orthopaedic Surgery, School of Medicine, University of Michigan, Ann Arbor, Michigan, United States of America; Università degli Studi della Campania, ITALY

## Abstract

Insufficient and delayed fracture healing remain significant public health problems with limited therapeutic options. Phosphoinositide 3-kinase (PI3K) signaling, a major pathway involved in regulation of fracture healing, promotes proliferation, migration, and differentiation of osteoprogenitors. We have recently reported that knock-in mice with a global increase in PI3K signaling (gCbl^YF^) show enhanced femoral fracture healing characterized by an extraordinary periosteal response to injury. Interestingly, of all growth factor receptors involved in fracture healing, PI3K directly binds only to PDGFR. Given these findings, we hypothesized a PDGFR-PI3K interaction is necessary for mediating robust periosteal cell activation following fracture. In this study, we isolated primary periosteal cells from gCbl^YF^ mice to analyze cross-talk between the PDGFRβ and PI3K signaling pathways. We found PDGFRβ signaling contributes to robust Akt phosphorylation in periosteal cells in comparison with other growth factor signaling pathways. Additionally, we performed femoral fractures on gCbl^YF^ mice with a conditional removal of PDGFRβ in mesenchymal progenitors using inducible alpha smooth muscle actin (αSMA) CreER^T2^ mice. Our studies showed that depletion of PDGFRβ signaling within these progenitors in the early phase of fracture healing significantly abrogates PI3K-mediated periosteal activation and proliferation three days after fracture. Combined, these results suggest that PDGFRβ signaling through PI3K is necessary for robust periosteal activation in the earliest phases of fracture healing.

## Introduction

Fracture repair involves complex interactions between cell lineages under the spatiotemporal control of growth factors and cytokines [1, 2]. A reported 5–10% of long bone fractures result in nonunion, and in certain populations such as the elderly, diabetics, and smokers, the incidence is significantly higher [3]. There remain limited pharmaceutical options in clinical practice for patients who experience delayed union or nonunion. While cell therapies are one possible approach to overcome this problem, developing a deeper understanding of the mechanisms that regulate the initial activation and expansion of periosteal cells will lead to improved therapeutic approaches. Our recent work has led us to become very interested in understanding the mechanisms required for the initial activation of periosteal cells in response to the fracture insult, which remain poorly defined.

Mesenchymal stem cells (MSCs) from various sources are required during the bone regeneration process, as they differentiate toward the osteochondral lineage and contribute to new bone formation [4]. Further research on MSC proliferation, differentiation, and migration is vitally important to the skeletal biology field, as they can be used for a variety of applications in the treatment of bone diseases [5]. Bone marrow-derived stromal cells (BMSCs) have historically been viewed as the canonical “stem cell” of the adult skeleton [6]. The field of bone biology is becoming increasingly aware of the critical importance of a source of progenitors within the periosteum, a dense connective tissue on the outer surface of bones. The inner, cellular cambium layer of the periosteum is a major source of progenitors, especially within the context of skeletal repair. Upon acute injury, progenitors harbored within the periosteal layer undergo major expansion in the areas immediately flanking the fracture site [7, 8]. This proliferation of periosteal cells flanking the fracture site in the early phase of healing is termed periosteal activation, and this cellular expansion post-injury is critical for optimal fracture healing [7].

Phosphoinositide 3-kinase (PI3K) signaling is a major pathway activated through receptor tyrosine kinases (RTKs) and G-protein coupled receptors [9]. The major downstream effects of the PI3K signaling pathway are mediated through the serine/threonine-specific protein kinase Akt, which is heavily involved in proliferation, differentiation, and survival of several cell types, including osteoblasts [10–12]. PI3K has established implications in osteogenesis, skeletal remodeling, and commitment to the osteogenic lineage [13]. We have recently reported that knock-in mice with a global increase in PI3K signaling (gCbl^YF^) show enhanced femoral fracture healing characterized by an extraordinary periosteal response, visible one to three days post-fracture [14]. This reported increase in periosteal thickness during mesenchymal progenitor activation correlates with an increase in osterix (Osx) and alkaline phosphatase (ALP) -positive bone lining cells. However, the growth factors that mediate this extraordinary periosteal activation are unknown.

Platelet-derived growth factor (PDGF) is a potent mitogen for mesenchymal progenitors [15–17], and is released by platelets and macrophages at fracture sites during the initial inflammation [18]. There are five members of the PDGF family (PDGF-AA, AB, BB, CC and DD). PDGFs signal through homodimers or heterdimers of receptor tyrosine kinases PDGFRα and PDGFRβ [19]. PDGF-BB is considered the most universal of the PDGF family ligands, as it can activate all receptor isoforms. Although PDGF has been approved for limited clinical applications in the context of orthopedics [18], its effects on osteogenic differentiation and bone formation are not fully understood. Interestingly, of the major growth factor receptors involved in fracture healing, PI3K can only directly bind PDGF-receptors (PDGFRs). Additionally, it has been recently published that a majority of murine periosteal cells post-fracture express the homodimer PDGFRβ or the heterodimer PDGFRα/β, while few express PDGFRα alone [20]. While a direct interaction between PI3K and PDGFRs is critical for skeletal development [21–23], this requirement in the context of periosteal response to fracture healing has never been studied. We hypothesized that a PDGFRβ-PI3K signaling axis mediates robust periosteal cell proliferation in the initial fracture healing response.

## Materials and methods

### Mouse strains

Cbl, an adaptor protein, negatively regulates of PI3K activity by binding to the p85 regulatory subunit of PI3K. Mutation of the tyrosine 737 into phenylalanine in Cbl (*Cbl*^*Y737F*^) results in abrogation of the Cbl-p85 association [24]. To study the impact of this interaction, a global knock-in mouse model in which the tyrosine 737 was substituted to phenylalanine (Cbl^Y737F/Y737F^, henceforth gCbl^YF^ mice) was used, and mice were genotyped as previously reported [25]. We have reported that the loss of this binding ability allows for increased PI3K activity in osteoclasts, osteoblasts and periosteal cells, which has been confirmed previously [14, 26–28]. To target periosteal progenitor cells at the time of injury, we conditionally deleted PDGFRβ by crossing the αSMA-CreER^T2^ with PDGFRβ^FL/FL^ mice on the gCbl^YF^ background (Table 1). 10-week-old mice were used for *in vivo* fracture studies, and 7-week-old mice were used for *in vitro* cell isolation and culture experiments. Mice of equal age were all of comparable weight. For continuity with previous studies by our group [12,24], female mice were used for *in vivo* fracture experiments. Mice were maintained at standard conditions with food and water *ad libitum* under a 12 hour light cycle. This study was approved by the local Institutional Animal Care and Use Committee (IACUC) at UConn Health, and was conducted in accordance with the national legislation on protection of animals and the NIH Guidelines for the Care and Use of Laboratory Animals.

**Table 1:** Genotypes of mice used for *in vivo* fracture experiments.

Table 1.	Designation	Genotype	Treatment
**Experimental Mice**	Cre+	αSMA-CreER^T2^/+; PDGFRβ^FL/FL^; gCbl^YF^	Tamoxifen
**Control Mice**	Cre-	+/+; PDGFRβ^FL/FL^; gCbl^YF^	Tamoxifen

### Surgical procedure

We performed femoral fracture surgeries and assessed the subsequent periosteal response on day 3 post-fracture. Stabilized closed femoral fractures were performed as previously described [14, 29]. Briefly, mice were anesthetized using 4% isoflurane and a 26-gauge needle (BD) was hand-drilled into the medullary cavity of the left femur under the patella. The needle was removed and immediately replaced with a blunt pin (0.38mm). After X-ray confirmation of pin placement, femurs were fractured using an Einhorn three-point bending device and evaluated immediately using x-ray. Buprenorphine (0.1 mg/kg) was administered by subcutaneous injection just prior to the procedure, and twice a day for three days following the fracture for pain management. Samples were excluded from the study analysis in cases of multi-fracture, bent pins, and fracture placement that was not mid-diaphyseal. Mice were sacrificed three days post-fracture for analyses, as this time point is ideal for studying the periosteal response to the fracture insult.

### Histology

To generate frozen sections, intact contralateral and fractured femurs were fixed in 10% neutral buffered formalin for 5 days. Undecalcified bones were then soaked in 30% sucrose overnight, and embedded in Optimal Cutting Temperature media (OCT) (Thermo Fisher Scientific, Hampton NH). Frozen sections (9μm) were cut, hydrated in phosphate buffered saline (PBS), counterstained with 4', 6-diamidino-2-phenylindole (DAPI), and mounted for imaging. Frozen sections were viewed on a Leica fluorescent microscope. To detect ALP+ cells, hydrated frozen sections were stained using a modification of the ALP Leukocyte kit (86R-1KT) (Sigma, St Louis, MO) with Fast Blue-BB salt (F3378) for 10–15 minutes in dark. Sections were then rinsed in PBS and counterstained with DAPI, then mounted for fluorescent imaging. ALP+ periosteal thickness was measured and quantified manually using ImageJ software. Data of cambium thickness, periosteal thickness, and ALP+ periosteal thickness are presented as averages of four sites (1500 μm from fracture site) in each sample.

### Cell culture

Periosteal cells were isolated from femurs and tibias of 7–9 week old mice as previously described [29]. Briefly, muscle, ligaments, and connective tissue were dissected away from the bone carefully with a scalpel. Epiphyses were cut from the end of each bone and the marrow was flushed with a 26-gauge needle using complete media comprised of alpha-minimum essential medium (αMEM) (Sigma) supplemented with 10% heat inactivated fetal bovine serum (FBS) (Hyclone, Logan, UT, USA) and 1% penicillin/streptomycin. Periosteum was scraped from flushed, hollow bones into PBS and digested enzymatically for one hour in an orbital shaker (0.05% collagenase P, 0.2% hyaluronidase in PBS). Complete media was added to stop enzymatic digestions and cells were filtered through a 40μm mesh strainer (Fisher Scientific) to form a single cell suspension and remove debris. Cells from 3–4 mice were plated in 6cm tissue-culture plates and grown in 5% oxygen for 4 days before transfer to normoxia (20% oxygen). To remove PDGFRβ *in vitro*, we passaged cells and performed an adenovirus-mediated infection using AdCre/AdGFP (300 MOI) (Vector Biolabs) at day 4 of cell culture (4x10^5^ cells/well in 6-well plates) on cells from PDGFRβ floxed mice. Viral media was replaced with complete media after 24 hours of infection.

### Quantitative RT-PCR

Total RNA was isolated using a Trizol (Invitrogen) extraction method and cDNA synthesized using Superscript III cDNA synthesis kit (Invitrogen). qRT-PCR reactions were performed on a Bio-Rad real-time PCR system using SYBR Green PCR reagents. GAPDH (sequence: F: 5’- AGGTCGGTGTGAACGGATTTG-3’, R: 5’- TGTAGACCATGTAGTTGAGGTCA-3’) was used as the housekeeping gene to normalize the expression level of PDGFRβ (sequence F: 5′-GATCCGCTCCTTTGATGATC-3′, R: 5′-GTCTCACACTTGCATGCCAG-3′).

### Western blotting

To analyze protein expression, periosteal cells were lysed in modified RIPA buffer and clarified total cell lysate was electrophoresed on 8% or 10% SDS-PAGE as described previously [26, 30]. Western blots were probed with the indicated antibodies. In some experiments, cells were kept in 0.1% FBS for 1–2 h, and were then treated with growth factors (e.g. FGF or PDGF-BB, 10ng/mL), PDGFR inhibitor (SU16f, 5μM), or a small molecule activator (SC79, 4μg/mL) for 30 minutes prior to preparation of cell lysates. The amounts of proteins in individual bands were quantified by using Odyssey Infrared Imaging Systems software 2.1 (LICOR Biosciences, Lincoln, NE) as previously reported.

### Flow cytometry

Periosteal cells were isolated and digested into single cell suspension as described above from both intact contralateral and fractured femurs three days post-fracture. After red blood cell lysis in ammonium-chloride-potassium buffer, approximately 2–4×10^6^ cells per/mouse were collected in cell staining media containing HEPES and 2% heat inactivated FBS in Hanks Balanced Salt Solution (HBSS). Cells were incubated with pacific blue-conjugated anti-CD45, pacific blue-conjugated anti-Ter119, pacific blue-conjugated anti-CD31, A700-conjugated anti-Sca1, PerCP-Cy5.5-conjugated anti-CD105, PE-conjugated anti-CD140b (PDGFRβ), FITC-conjugated anti-CD29, and APC-conjugated anti-CD140a (PDGFRα) (eBioscience, San Diego, CA, USA), at a 1:400 dilution in staining media for 1 hour on ice. Cells were washed in staining media and stained for live-dead with Zombie UV (Biolegend, San Diego, CA, USA) and analyzed using BD-LSRII (BD Biosciences, San Jose, CA, USA). Following gating for live-dead and background staining using isotype controls, the percentage of CD45− CD31− Ter119− Triple Negative (TN) cells expressing periosteal markers such as Sca1, CD105, and CD29 was examined. Levels of PDGFRα- and β-positive cells in these gatings were examined. Data is presented as the percentage of mesenchymal cells expressing PDGFRα, PDGFRβ, or PDGFRα/β.

### *In vitro* proliferation assay

To determine if PDGFRβ removal affected periosteal cell proliferation *in vitro*, we cultured primary periosteal cells from PDGFRβ^FL/FL^; gCbl^YF^ mice. Passaged periosteal cells were seeded at 4x10^5^ cells/well in 6-well plates and incubated with growth medium overnight. Medium was changed to DMEM, 0.5%FBS, 1%P/S and AdCre or AdGFP (300 MOI) overnight to recombine the floxed PDGFRβ alleles. GFP expression in AdGFP-treated wells was used to confirm recombination. Cells were treated with a combination of PDGF-BB (10ng/mL) and 5-ethynyl-2’-deoxyuridine (EdU, 10μM) for four hours prior to harvest by trypsinization, and stained using the Click-iT EdU Pacific Blue kit (Invitrogen) following manufacturer’s instructions. The number of live, single cell, EdU+ cells was determined by flow cytometry on a LSR II flow cytometer (Becton-Dickinson) and analysis was performed using DIVA and FlowJo software.

### *In vivo* proliferation assay

To determine if PDGFRβ removal affected periosteal cell proliferation *in vivo*, we performed a flow cytometry-based *in vivo* proliferation assay. Mice underwent femoral fracture with tamoxifen induction to induce PDGFRβ deletion in αSMA+ periosteal progenitors. 100μL EdU in DMSO (10mM) was injected two days post-fracture. Intact and fractured femurs were isolated three days post-fracture, and periosteal cells isolated from the intact bones or from the fracture site and digested into a single cell suspension, as described in methods above. After red blood cell lysis as described above, cells from individual mice were collected in staining media containing HEPES and 2% heat inactivated FBS in Hanks Balanced Salt Solution (HBSS). Periosteal cells were incubated with pacific blue-conjugated anti-CD45, pacific blue-conjugated anti-Ter119, pacific blue-conjugated anti-CD31, and PE-conjugated anti-CD140b, (eBioscience, San Diego, CA, USA), at a 1:400 dilution in staining media for 1 hour on ice. Cells were washed in staining media and stained for live-dead with Zombie UV (Biolegend, San Diego, CA, USA), then stained using the Click-iT EdU Alexa Fluor 488 kit (Invitrogen) following manufacturer’s instructions. Cells were analyzed on a BD-LSRII (BD Biosciences, San Jose, CA, USA). Following gating for live-dead and background staining using isotype controls, the percentage of CD45− CD31− Ter119− Triple Negative (TN) cells expressing PE (CD140b) and Alexa Fluor 488 (EdU+, proliferating cells) was examined. Analysis was performed using DIVA and FlowJo software.

### Statistical analysis

Results were analyzed using Student’s t-test. Data are presented as mean ± SD. *p*-values < 0.05 were considered statistically significant.

## Results

### Expression of PDGF-receptors in periosteum and fracture repair

**Fig 1:**
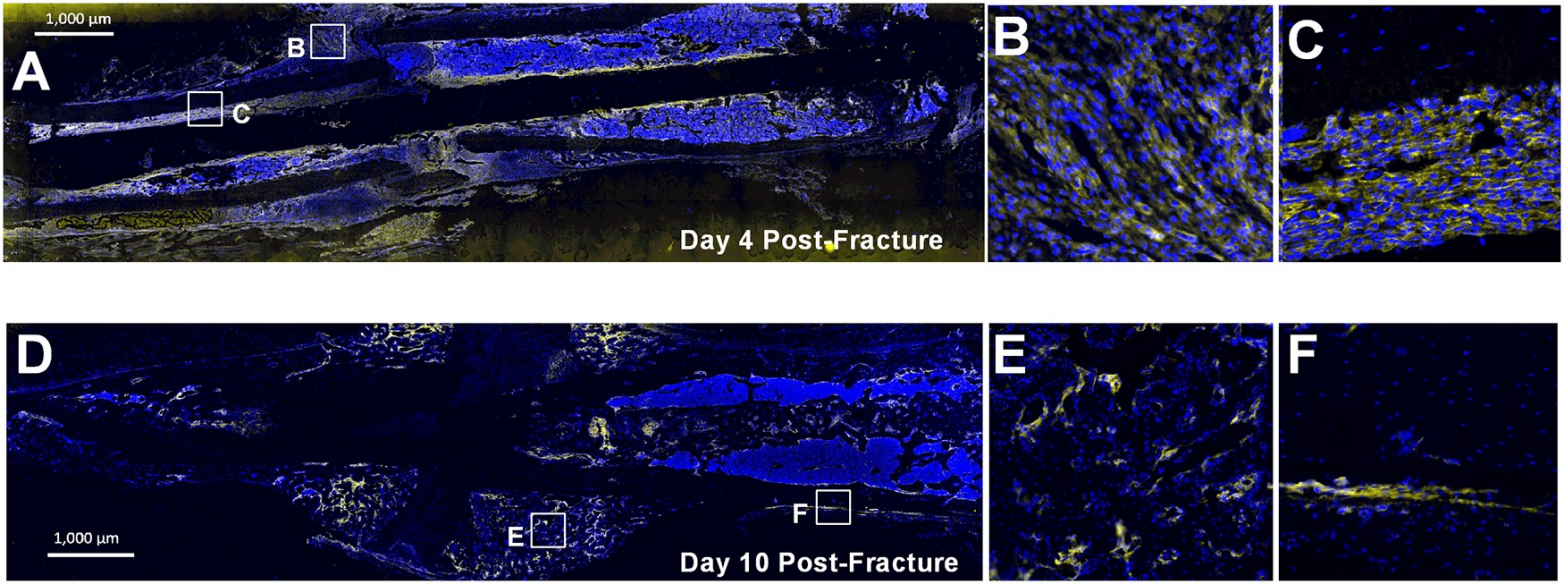
PDGFRβ is highly expressed in the periosteum post-fracture. (A) 10x stitched tile scan image and (B,C) 100x magnification of selected locations (boxes) showing expansive PDGFRβ expression in the periosteum day four post-fracture. (D) 10x stitched tile scan image and (E,F) 100x magnification of non-cartilaginous areas of the fracture callus day ten post-fracture. B,E are located close to the fracture site, while C, F are distal to the fracture site showing activated periosteum. Blue = DAPI. Yellow = PDGFRβ immunostaining. Scale bar indicates 1,000μm.

Growth factor (GF) levels increase at the onset of fracture, as various cells and platelets actively release GFs to stimulate bone repair. Selective binding of GFs to RTKs promotes the downstream effects of PI3K signaling in osteoprogenitors. However, it remained unclear, which specific GFs are able to most effectively activate the PI3K pathway in periosteal progenitors. The effects of platelet-derived growth factor (PDGF) are mediated through the specific RTKs PDGFRα and PDGFRβ, which form both homodimers and heterodimers [19]. By flow cytometry analysis, we found that after *in vitro* expansion, 75% of primary periosteal cells express PDGFRβ, while only 6% express both PDGFRα and β (S1A Fig). To confirm these results, we additionally examined the expression of PDGFRβ at different stages of the fracture healing process using immunostaining (Fig 1). At day four post-fracture (an early fracture-healing time point), PDGFRβ is highly expressed in activated periosteum flanking the site of injury (Fig 1A and 1B) as well as within the bone marrow compartment (Fig 1C). We also found PDGFRβ to be broadly expressed in cells of the fracture callus day ten after fracture, excluding cartilaginous areas (Fig 1D–1F).

### Effect of PI3K on PDGF-receptor expression and phosphorylation

**Fig 2:**
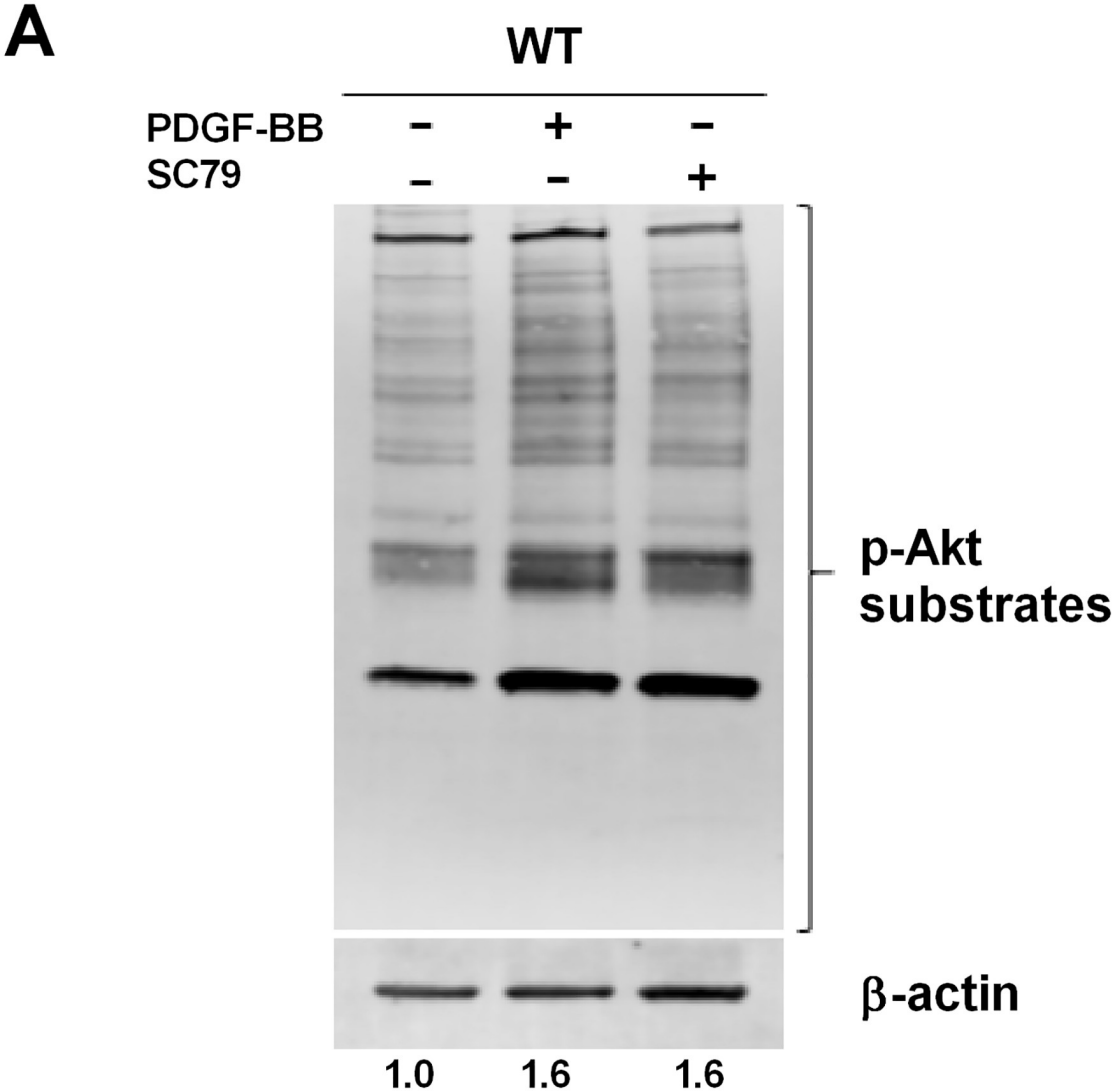
PDGF/PDGFR binding mediates downstream PI3K signaling activity in periosteal cells. (A) WT periosteal cells were serum starved for 1 hour, then were left untreated or were treated with SC79 (4μg/mL). Cells were also treated with PDGF-BB (10ng/mL) to target all isoforms of the PDGFR-family. Total cell lysate (40 μg) was electrophoresed on 8% SDS-PAGE. The blot was probed using anti-Akt substrates antibody (*top*), and then stripped and reprobed with anti-β-actin (*bottom*) to verify equal loading of protein. The ratio of phosphorylated and total protein is indicated. A representative of two experiments is shown.

Robust PDGFRβ expression in the periosteum and fracture callus confirmed an important role for PDGF in the context of skeletal regeneration. Binding of PDGF to PDGFRs induces a signaling cascade initiated by auto-phosphorylation of receptors, which subsequently activate PI3K/Akt signaling [31]. To explore this further, we characterized the effects of PDGF/PDGFRβ binding on downstream PI3K activity within periosteum. SC79 is a small molecule, which specifically binds to the Pleckstrin homology domain of Akt, and enhances Akt activity in various physiological conditions [32, 33]. Treatment of wild-type periosteal cells with PDGF-BB induced Akt phosphorylation to the same extent as treatment with SC79 (Fig 2). This confirmed that, in periosteal cells, PDGF binding to its receptors very efficiently activates PI3K-AKT signaling.

**Fig 3:**
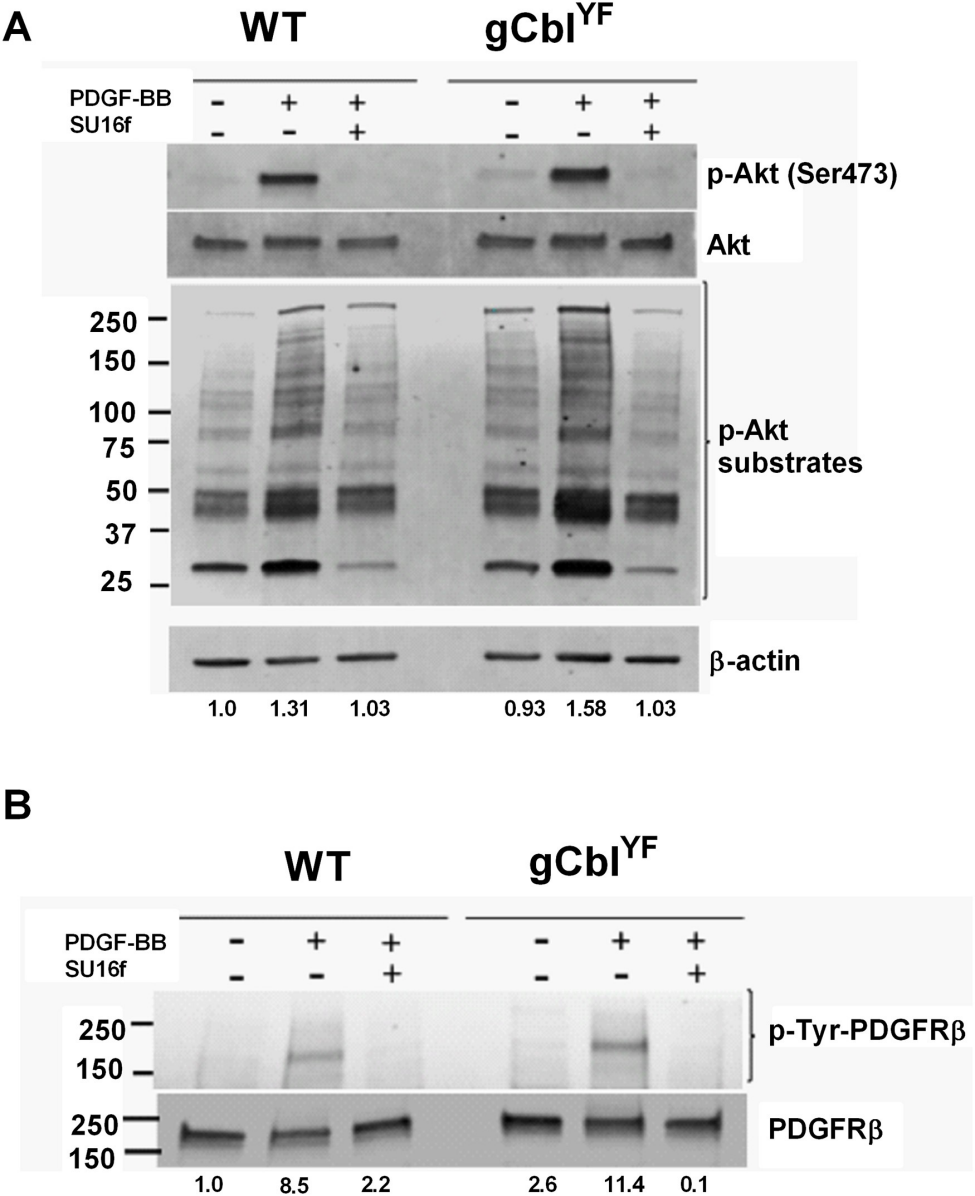
PI3K upregulation sensitizes periosteal cells to the effects of PDGF. Periosteal cells isolated from WT or gCbl^YF^ mice were serum starved for 1–2 hours and then treated with PDGF-BB (10ng/mL) or SU16f (5μM), a PDGFR inhibitor, for 30 minutes. (A) Total cell lysate (40 μg) was electrophoresed on 10% SDS-PAGE. Western blots were probed with anti phospho-Akt antibodies and anti-Akt antibodies. Another set of blots were probed with anti-Akt substrate antibody (*top*), and then stripped and reprobed with anti-β-actin antibodies (*bottom*) to verify equal loading of protein. The ratio of phosphorylated and total protein is indicated at the bottom of each lane. (B) The blot was probed using anti-phospho-tyrosine antibody (*top*), to determine phosphorylation of the PDGFR, and then stripped and reprobed with anti-PDGFRβ (*bottom*) to verify equal loading of protein. The ratio of phosphorylated and total protein is indicated. A representative of two experiments is shown.

Our previous studies using a mouse model of globally upregulated PI3K activity (gCbl^YF^) revealed a remarkable periosteal response to fracture; however, these studies had not investigated changes in GF binding responses in the context of upregulated PI3K signaling activity. Therefore, we were interested in determining how PI3K upregulation modulates the effects of PDGF, and whether this provided an even more robust response to PDGF stimulation. We isolated periosteal cells from gCbl^YF^ mice and cultured them to confluence alongside wild-type cells. PDGF-BB treatment augmented phosphorylation of Akt and Akt substrates containing the RXXS*/T* motif in gCbl^YF^ periosteal cells compared to wild-type (Fig 3A). In contrast, FGF, an important GF in fracture healing, resulted in insignificant changes in pAkt in cultured periosteal cells (S2 Fig). Phosphorylation of Akt substrates was significantly reduced when cells were treated with the PDGFRβ inhibitor SU16f, demonstrating a PDGF-specific effect (Fig 3A). Treatment of wild-type of periosteal cells with PDGF-BB resulted in enhanced phosphorylation of PDGFRβ compared to the untreated cells (Fig 3B). Moreover, treatment of gCbl^YF^ periosteal cells with PDGF-BB resulted in ~3 times more phosphorylation of PDGFRβ compared to wild-type treated cells. Exposure of both cell types to SU16f inhibited PDGFRβ phosphorylation. Together, these results indicate that upregulated PI3K activity in periosteal cells allows for augmented effects of PDGF/PDGFR signaling.

To further evaluate this response in gCbl^YF^–derived periosteal cells, we performed qRT-PCR analysis. Our data show that periosteal cells derived from gCbl^YF^ mice have a 20-fold increase in PDGFRβ expression compared to wild-type cells during the proliferative phase of cell culture (S3A Fig). Flow cytometry analyses on these cells confirmed significantly increased double-positive PDGFRα/β expression in gCbl^YF^ periosteum compared to wild-type (S3B Fig). These data indicate that the response to PDGF seen in the context of upregulated PI3K signaling could be a result of increased receptor availability.

### PDGFRβ is required for PI3K-induced periosteal activation during fracture healing

We have previously reported that gCbl^YF^ mice show an enhanced periosteal response to fracture, characterized by increased proliferation of periosteal cells and Osterix expression within the periosteal layer day three post-fracture [14]. This time point is ideal for studying the periosteal response to injury.

**Fig 4:**
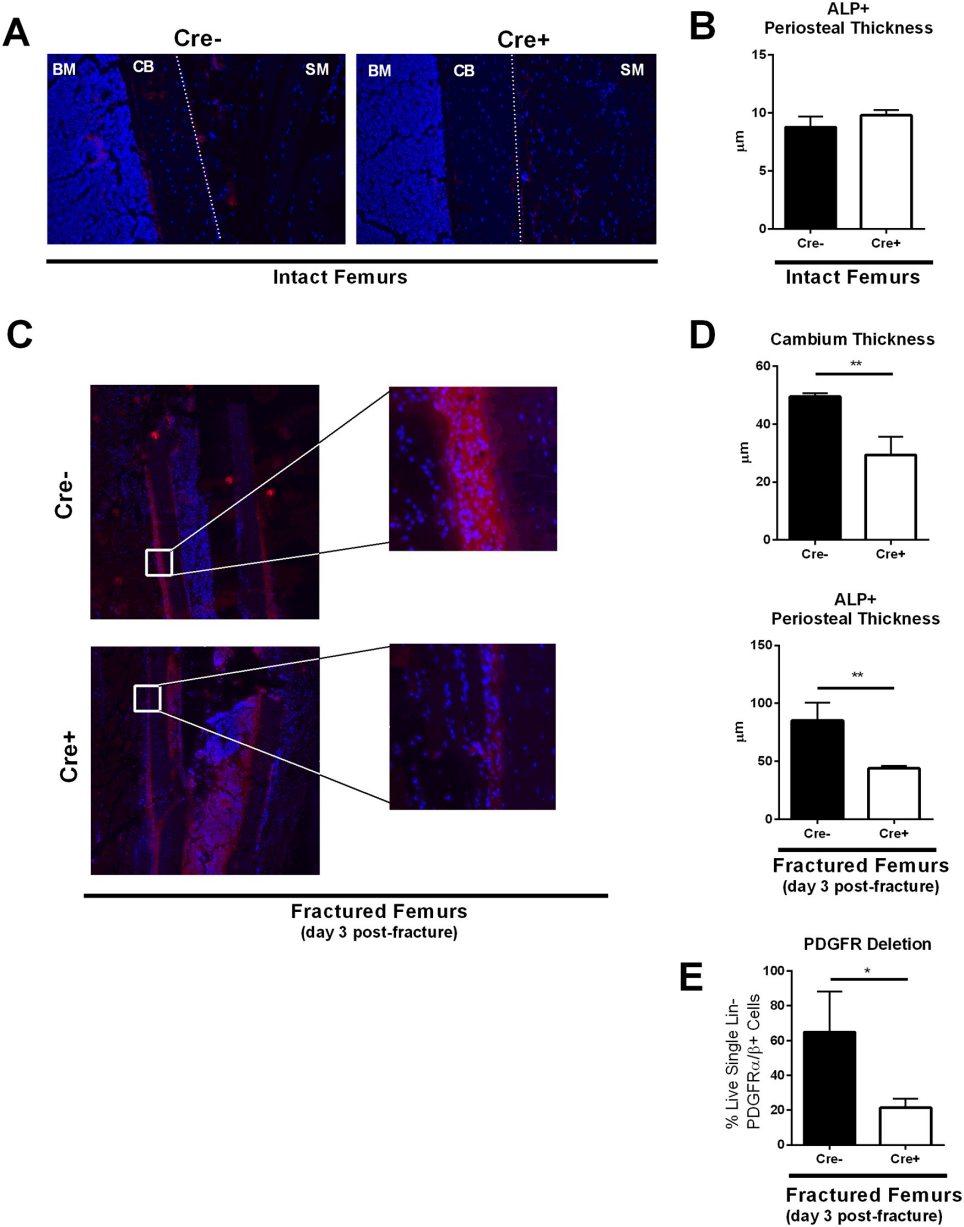
αSMA-Cre-mediated removal of PDGFRβ in periosteal progenitors after bone injury dramatically reduces the periosteal response to fracture seen in gCbl^YF^ mice. (A) Representative images of the periosteal thickness and ALP activity in intact bones. 10x magnification. Blue = DAPI, red = ALP activity stain. BM = bone marrow, CB = cortical bone, dotted lined = periosteum, SM = skeletal muscle. N = 3–4 biological replicates per group. (B) Quantification of ALP activity stain thickness (μm) in intact bones. Tamoxifen (75μg/g body weight) for conditional deletion of PDGFRβ was administered at days 0 and 1 prior to sacrifice. N = 3–4 biological replicates per group. (C) Fractured femurs from Cre- and Cre+ mice at 10x magnification (Table 1). Representative images of the periosteal thickness and ALP activity 1500μm from the fracture site at day 3 post-fracture. Blue = DAPI, red = ALP activity stain. CB = cortical bone, SM = skeletal muscle. Tamoxifen (7.5μL/gram) for conditional deletion of PDGFRβ was administered at days 0 and 1 post-fracture. N = 3–4 biological replicates per group. (D) Quantification of cambium layer thickness and ALP activity stain thickness (μ). Each value represents the average of four measurements at different sites 1500μm away from the fracture site in one sample. N = 3 biological replicates per group. **p<0.005 (E) To confirm PDGFR deletion in our mouse model, we isolated periosteum from the fracture site day three post-fracture from both experimental (Cre+) and control (Cre-) groups. Cells were digested into single cell suspension and stained for flow cytometry analyses. We analyzed the levels of PDGFRα and PDGFRβ in Lin- (CD45- TER119- CD31-) cells to validate our inducible mouse model. N = 3 *p<0.05.

To understand the role of PDGF in PI3K-mediated periosteal activation, we generated a mouse model to delete PDGFRβ expression in periosteal progenitors upon injury. We crossed a PDGFRβ^FL/FL^ mouse with the inducible αSMA-CreER^T2^ on the gCbl^YF^ background, and generated closed, stabilized femoral fractures. αSMA-CreER^T2^ is expressed in mesenchymal progenitors during fracture healing, and has been used in multiple studies to target the periosteal layer during skeletal repair [34, 35]. Experimental mice and controls (Table 1) were injected with tamoxifen one day prior to and the day of fracture to induce removal of PDGFRβ in periosteal progenitor cells. In intact limbs from both experimental and control mice, there was no significant difference in periosteal thickness or alkaline phosphatase (ALP) activity within the periosteal layer (Fig 4A and 4B).

Following fracture of control mice on the gCbl^YF^ background, we saw a robust periosteal thickening three days after fracture using histological analyses (Fig 4C). This periosteal activation was also paralleled by intense ALP activity in this tissue layer (Fig 4C), correlating with data we have previously published. However, tamoxifen-induced removal of PDGFRβ in our experimental mice on the gCbl^YF^ background resulted in a dramatic loss of periosteal thickness flanking the fracture site compared to controls (Fig 4C). The cellular cambium layer of the periosteum demonstrated significantly reduced expansion, as well as decreased ALP activity in the periosteal layer compared to controls (Fig 4D). The conditional deletion of PDGFRβ in periosteum day three post-fracture was confirmed using flow cytometry (Fig 4E). These results strongly implicate PDGF/PDGFRβ in the mechanisms that drive periosteal activation in this early phase of fracture healing in the gCbl^YF^ mice.

**Fig 5:**
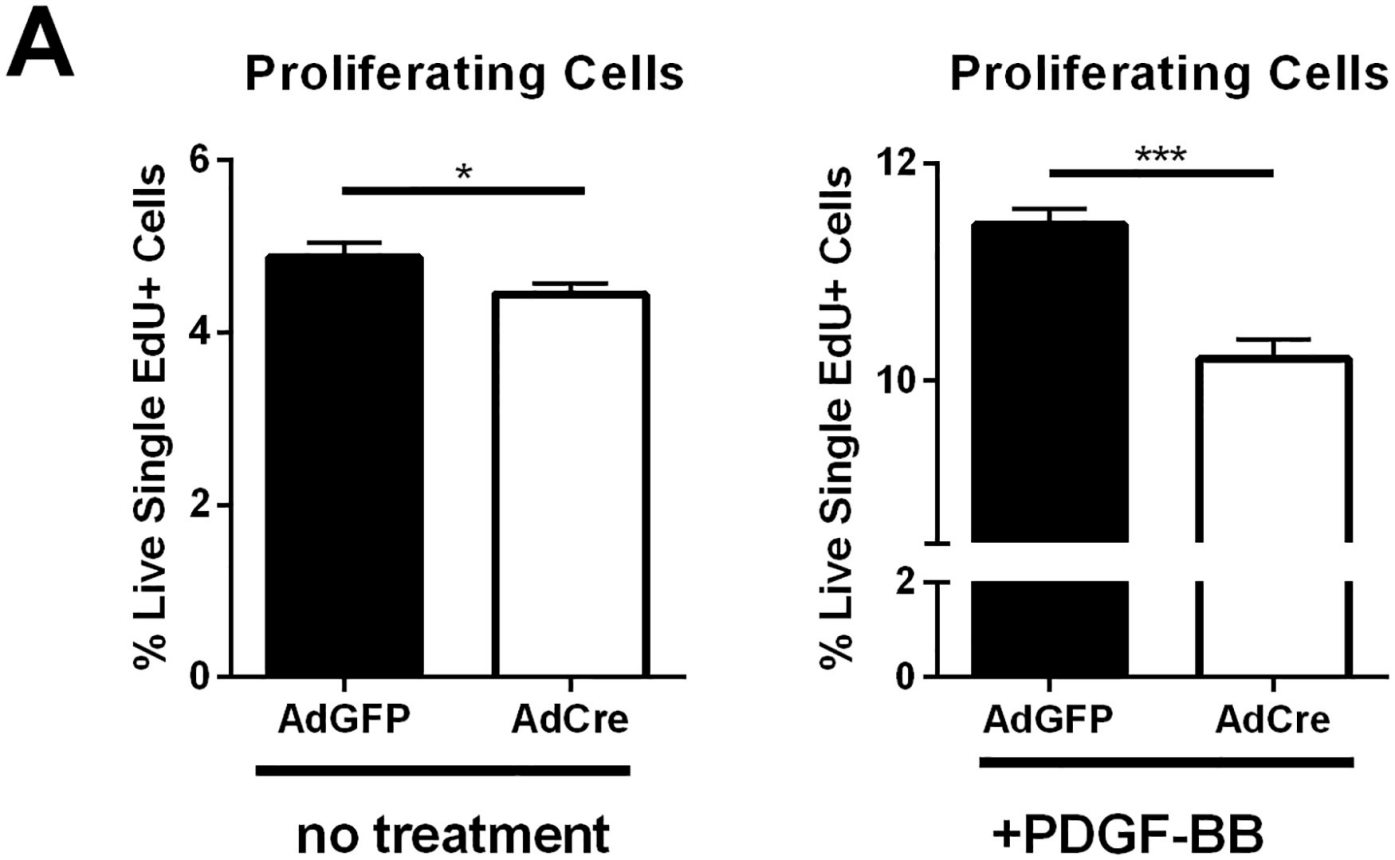
PDGFRβ is required for robust periosteal proliferation *in vitro* in cells derived from gCbl^YF^ mice. (A) Periosteal cells isolated from PDGFR^FL/FL^; gCbl^YF^ mice were cultured for four days, and then serum starved and infected with AdCre or AdGFP overnight (MOI = 300). Cells were treated with EdU (10mM) for four hours prior to isolation and staining. Flow cytometry analysis of EdU+ periosteal cells *in vitro* shows significantly less proliferation in periosteal cells following AdCre-mediated deletion of PDGFRβ on the gCbl^YF^ background, without PDGF addition. N = 3 *p<0.05 (B) Periosteal cells isolated from PDGFR^FL/FL^; gCbl^YF^ mice were cultured for four days, and then serum starved and infected with AdCre or AdGFP overnight (MOI = 300). Cells were then treated with PDGF-BB and EdU (10mM) for four hours prior to isolation and staining. Flow cytometry analysis of EdU+ periosteal cells *in vitro* shows significantly less proliferation in periosteal cells treated with PDGF-BB following AdCre-mediated deletion of PDGFRβ on the gCbl^YF^ background. N = 3 ***p<0.0005.

**Fig 6:**
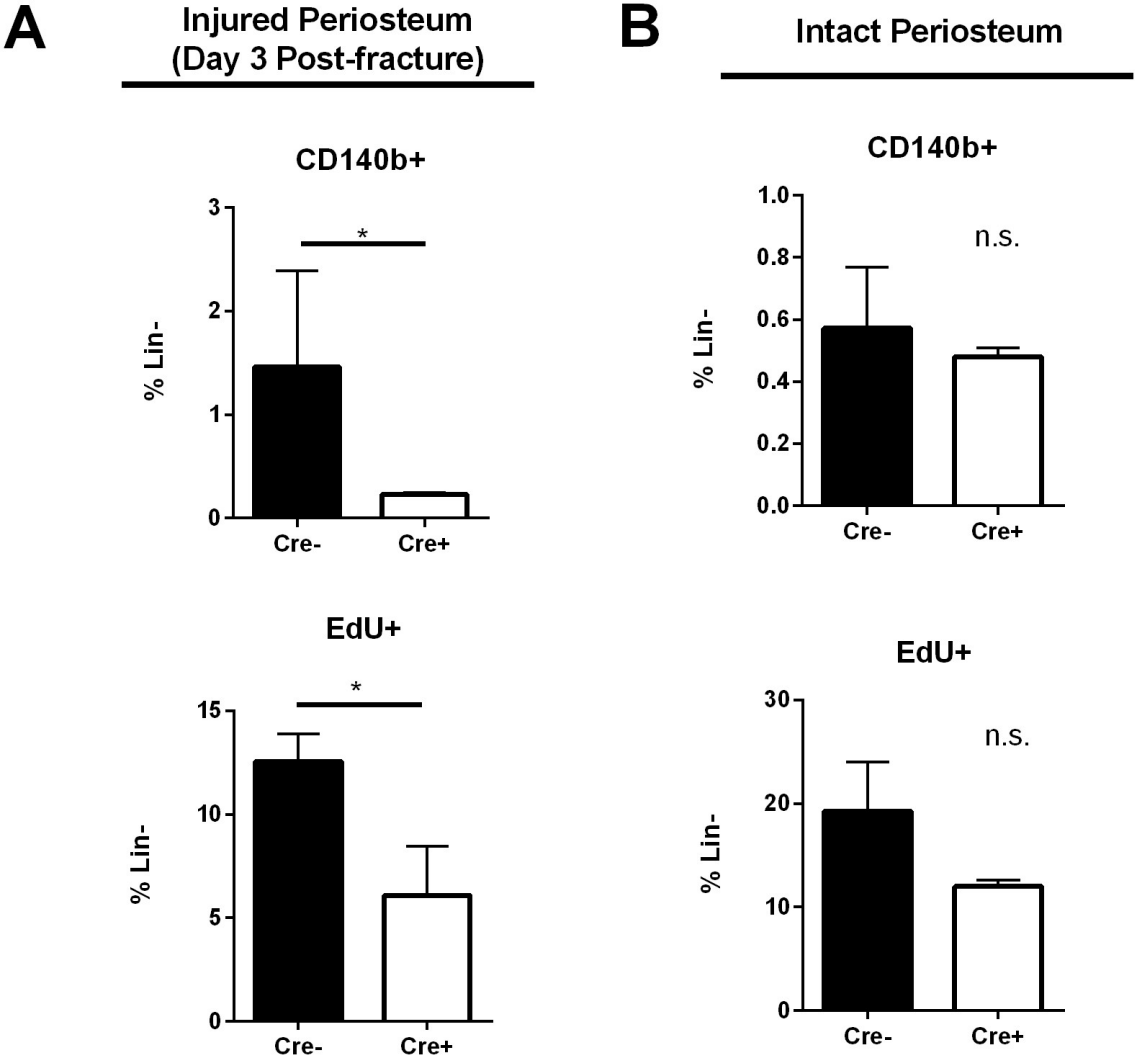
PDGFRβ is required for robust periosteal proliferation *in vivo* after fracture in mice on the gCbl^YF^ background. (A) αSMA-CreERT2/+; PDGFR^FL/FL^; gCbl^YF^ mice and PDGFR^FL/FL^; gCbl^YF^ controls underwent femoral fracture. Tamoxifen (75μg/g body weight) for conditional deletion of PDGFRβ was administered at days 0 and 1 prior to sacrifice. EdU (10mM) was injected one day prior to sacrifice. Periosteal cells were isolated from the fracture site and subjected to flow cytometry analyses for proliferating cells and cells expressing PDGFRβ. N = 3–4 biological replicates per group. *p<0.05 (B) Periosteal cells were isolated from intact contralateral femurs and subjected to flow cytometry analyses for proliferating cells and cells expressing PDGFRβ. N = 3–4 biological replicates per group. *p<0.05.

To determine an underlying mechanism for these results, we analyzed the proliferation potential of gCbl^YF^ periosteal cells with either intact or lineage-deleted PDGFRβ. Our previously published data indicates that proliferation is remarkably increased in gCbl^YF^ periosteal cells post-fracture [14], and PDGF has a known proliferative effect in mesenchymal cells. We found that removal of PDGFRβ in periosteal cells on the gCbl^YF^ background significantly reduces the proliferative capacity of these cells when stimulated with PDGF-BB *in vitro* (Fig 5A and 5B). We additionally tested the effect of PDGFRβ/PI3K signaling interaction on periosteal proliferation *in vivo*. Periosteal cells from mice with conditional deletion of PDGFRβ in αSMA+ cells post-fracture exhibited significantly reduced proliferation following the surgical procedure compared to Cre-negative controls (Fig 6A). Periosteal cells with a conditional deletion of PDGFRβ did not show a significant difference in proliferative capacity in the absence of a fracture insult (Fig 6B). Combined, these results suggest that PI3K-induced periosteal proliferation and expansion after fracture requires a PDGF/PDGFRβ interaction.

## Discussion

The role of PI3K signaling in the periosteum has been hitherto unexplored in the context of the osteogenic response of periosteal cells during fracture healing. Wnt, Notch, BMP, and other major pathways have all been implicated in influencing the periosteal response to fracture, and PI3K forms a major node downstream of these signaling pathways [36]. To date, a vast majority of studies on the effects of PI3K in governing the differentiation and function of bone cells have been done using knockout mouse models. The effect of PI3K signaling in fracture studies has been analyzed indirectly and only in mature osteoblasts by deleting PTEN [37]. These studies did not evaluate the requirement of PI3K activation in periosteal precursors during callus formation. Thus, to our knowledge there are no previous studies that interrogate the direct impact of increased PI3K/Akt activity in periosteal cells during the course of fracture healing. Understanding how PI3K/Akt activity regulates periosteal expansion in response to PDGF may provide valuable insights into developing new and improved therapeutics to treat fractures. Of particular interest would be small molecules that regulate Class IA PI3Ks and Akt.

A robust periosteal activation and expansion post-fracture, such as that seen in gCbl^YF^ mice, provides a greater number of osteochondral progenitors to the injury site that can contribute to the fracture healing process. In the present study, we determined a requirement for intact PDGF/PDGFRβ signaling in the context of this PI3K-mediated periosteal activation.

We initially found that a majority of periosteal cells express PDGFRβ in culture, and that PDGFRβ is broadly expressed within cells of the fracture callus. In isolated periosteal cells, PDGFRα expression was minimal compared to that of PDGFRβ, similar to findings we have recently reported [38]. These results strengthened our hypothesis that PDGFRβ is a critical receptor that mediates periosteal activation.

We show that PDGF treatment of periosteal cells phosphorylates Akt substrates to the same extent as SC79, a small molecule activator of Akt. This further confirmed that PDGF/PDGFR signaling is a critical component of periosteal PI3K activity. Combined with our results that PDGF is able to induce phosphorylation of Akt to the greatest extent out of multiple growth factors, and knowledge of the literature on growth factor involvement in skeletal healing, we were confident in pursuing a PDGFRβ/PI3K signaling axis in the periosteum.

Periosteal cells isolated from gCbl^YF^ and WT mice showed no significant difference in the ability to phosphorylate PDGFRβ at basal conditions. Despite this, addition of PDGF-BB to cultures induced greater phosphorylation of PDGFRβ in gCbl^YF^ cells compared to WT. These findings indicate that upregulated PI3K activity, while having no effect on periosteal downstream signaling targets at baseline, may prime cells for signaling responses to specific growth factors.

To understand the implications of our results *in vivo*, we generated two groups of mice of the gCbl^YF^ background: our control with intact PDGFRs, and our experimental mice, with inducible αSMA-Cre-driven deletion of PDGFRβ. αSMA-CreER^T2^ is widely used to target skeletal progenitors following a fracture insult. In our study, we saw no change in either periosteal cambium thickness or ALP activity in intact limbs upon tamoxifen induction; this is consistent with data reported by other group, as αSMA marks a small population of periosteal cells (~1%) that becomes largely activated after a fracture insult [39, 40]. We next determined the requirement of PDGFRβ in mediating the robust periosteal response to fracture normally seen in the context of upregulated PI3K signaling. Using the αSMA-CreER^T2^ mouse model to target the periosteum after fracture, we observed a dramatic reduction in periosteal activation in those mice with temporal-spatial deletion of PDGFRβ. These results indicate that periosteal proliferation post-fracture is enhanced through the PI3K/Akt signaling pathway, but that this mechanism requires an intact PDGF/PDGFRβ interaction.

Our focus on PDGF-regulated signaling and PDGFRβ in particular is grounded in both our experimental results and the relevant literature. PDGF is a potent mitogen, and has been shown previously to affect proliferation and expansion of mesenchymal cells, both *in vitro* and *in vivo*. Massive amounts of this GF are released by platelets and macrophages during the earliest phases of fracture healing [18]. The importance of PDGFRβ signaling has been well-studied during development, and it is a potent regulator of mesenchymal stromal cell function [41–43]. In a conditional knock-in mouse model, Olson and Soriano [44] found that increased PDGFRβ signaling drives cell proliferation and opposes differentiation of vascular smooth muscle and pericytes, maintaining progenitor potential *in vivo*. Depletion of PDGFRβ in mesenchymal stromal cells also decreased the proliferation and migration response while promoting osteogenic differentiation [17]. Other growth factors, such as vascular endothelial growth factor (VEGF), have more specialized roles in fracture healing processes (e.g. angiogenesis and inflammatory cell recruitment), and we show in the present study that FGF does not mediate strong PI3K activity in periosteal cells. With the development of small molecules that can directly influence activity of PI3K or Akt, and potentially be used for treating non-healing fractures, it is imperative to know whether the PDGF-PI3K-Akt signaling axis is required for a periosteal response to bone injury.

As noted by Mele *et al*., the mobilization and activation of endogenous stem cells is an important field of study in the context of tissue regeneration [45]. Addition of PDGF at the site of skeletal or vertebral injury has been proposed as a possible therapy for bone healing and repair [46]. Our results suggest an activator of PI3K may enhance therapeutic outcomes by enhancing the downstream signaling effect of PDGF/PDGFR binding. One could even take advantage of the endogenous growth factors that are produced at the fracture site if the injured area is sensitized by a potent Akt activator SC79, bypassing the need for supra-physiologic dose of PDGF. SC79 has been shown to reduce the area of cortical infarct in a rat model of brain ischemia [33], and treatment with SC79 in rats enhanced femoral fracture healing by accelerating atrophic quadriceps recovery [47]. These studies combined indicate a promising future for Akt activators in the context of wound healing.

Our study has some minor limitations, most notably that there is no true marker of the periosteum for *in vivo* studies. The scope of this study is limited to periosteal progenitors, and we have not yet explored the possibility of a PDGFR/PI3K signaling axis in other progenitor compartments involved in fracture healing; however, the periosteum is known to be the major contributor to skeletal repair when compared to the bone marrow stroma and perivascular cells [48].

In conclusion, our study demonstrates that PDGFRβ signaling is necessary for robust periosteal activation in the earliest phases of PI3K-mediated fracture healing. We propose that PI3K signaling enhances the effect of PDGF in the fracture healing response, and this information can be harnessed to improve therapeutic options. A deeper understanding of the molecular mechanisms that mediate the periosteal injury response is critical to the development of new and more efficient therapies to reduce the incidence of delayed bone union and nonunion.

## Supporting information

S1 Fig A majority of periosteal cells express PDGFRβ. (A) Wild-type primary periosteal cells cultured to confluence were harvested for flow cytometric analysis and gated for live, single cell, Lin- (CD45- TER119- CD31-), PDGFRα/β+ populations.
(TIF)

S2 Fig FGF is not strongly implicated in PI3K-mediated periosteal activation after fracture. (A) Periosteal cells isolated from gCbl^YF^ mice were serum starved overnight and then treated with PDGF-BB (10ng/mL) or FGF2 (10ng/mL) for 30 minutes. Western blot was probed for the indicated antibodies.
(TIF)

S3 Fig Globally upregulated PI3K signaling in periosteal cells results in significantly upregulated PDGFRβ expression. (A) qRT-PCR results for *Pdgfrβ* expression in WT and gCbl^YF^ periosteal cells from three or seven days in culture. (B) Flow cytometry results for PDGFRα/β expression in WT and gCbl^YF^ periosteal cells harvested at confluence. Cells were gated for live, single cell, Lin- (CD45- TER119- CD31-), PDGFRα/β+ populations.
(TIF)

S1 Raw Images Raw blot images. Uncropped raw images of gels and blots appearing the in manuscript, with the corresponding figure number noted above each image.
(PDF)

S1 Minimal Data Set Values used to generate data presented in specific figures within the manuscript, with the corresponding figure number noted above each data set. (PDF)
