# Supplementary material for: A PDGFRβ-PI3K signaling axis mediates periosteal cell activation during fracture healing
Source: PLoS One. 2019 Oct 30;14(10):e0223846. doi: 10.1371/journal.pone.0223846 (PMC6821073; doi:10.1371/journal.pone.0223846)

Fig. 2A

p-Akt substrates β-actin

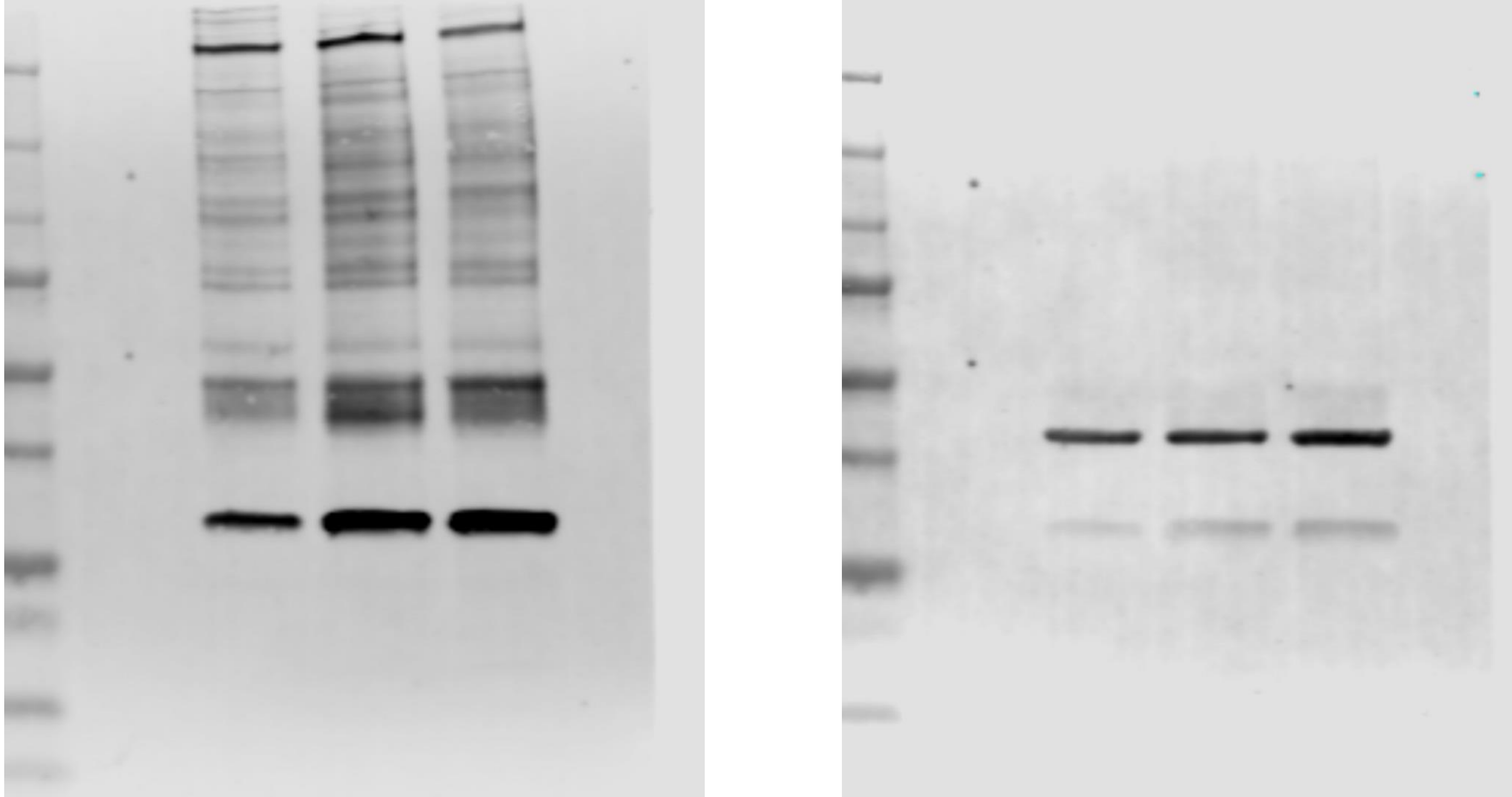

Fig. 3A

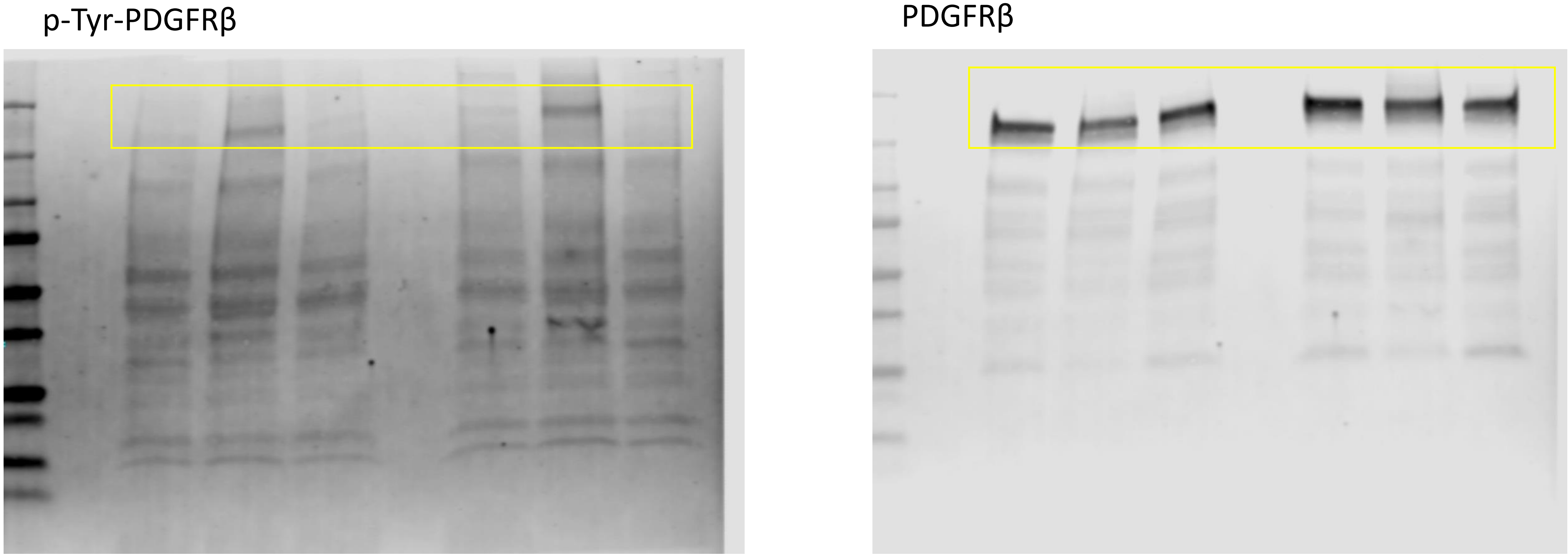

Fig. 3B (top)

p-Akt (Ser473)

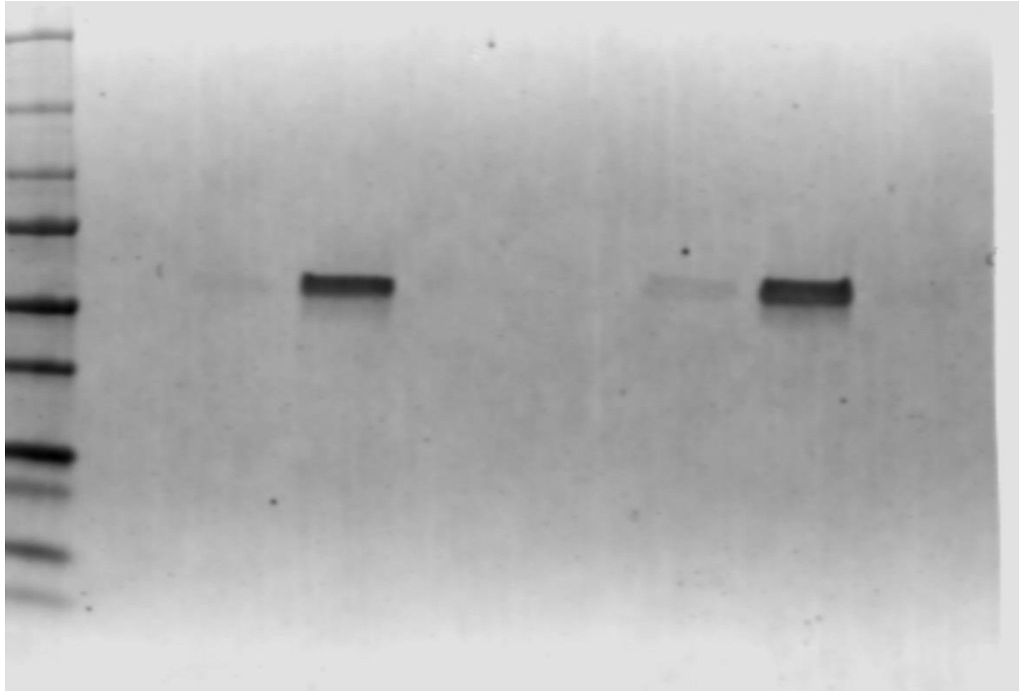

Akt

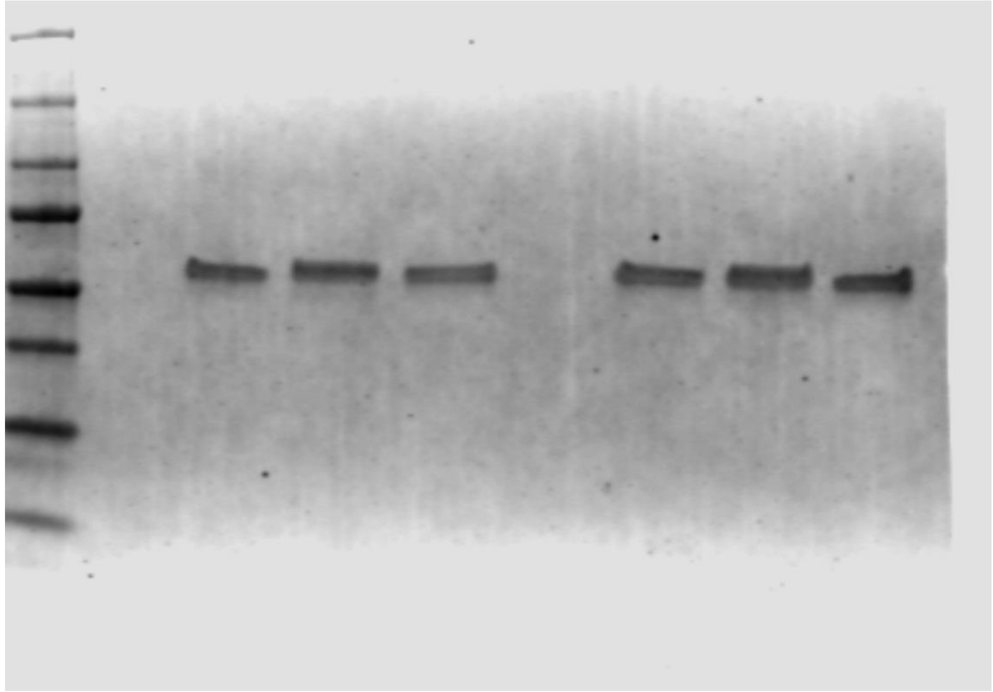

Fig. 3B (bottom)

p-Akt substrates β-actin

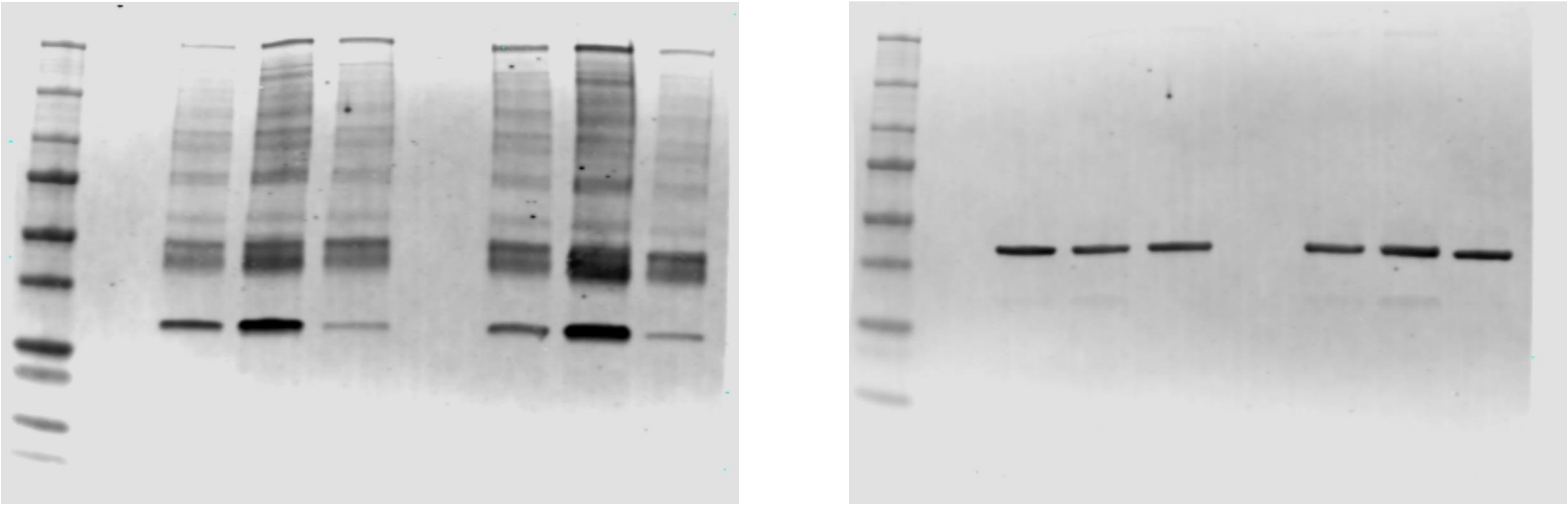

Supplement: S1 Raw Images — Uncropped raw images of gels and blots appearing the in manuscript, with the corresponding figure number noted above each image. (PDF) [file pone.0223846.s004.pdf]
