# Supplementary material for: A PDGFRβ-PI3K signaling axis mediates periosteal cell activation during fracture healing
Source: PLoS One. 2019 Oct 30;14(10):e0223846. doi: 10.1371/journal.pone.0223846 (PMC6821073; doi:10.1371/journal.pone.0223846)
Supplement: S1 Minimal Data Set — (PDF) [file pone.0223846.s005.pdf]

Minimal Data Set

Fig. 4B

ALP+ Periosteal Thickness*

Cre-	Cre+
7.98	9.28
9.82	9.99
8.45	10.15

* ALP+ periosteal thickness was measured and quantified manually using ImageJ software. ALP+ periosteal thickness are presented as averages of four sites (1500 µm from fracture site) in each sample, in 3 samples total.

Fig. 4D

Cambium Thickness*

Cre-	Cre+
49.65	24.25
50.67	34.9
48.29	34.59
	23.6

*Cambium thickness was measured and quantified manually using ImageJ software. ALP+ periosteal thickness are presented as averages of four sites (1500 µm from fracture site) in each sample, in 3-4 samples total.

ALP+ Periosteal Thickness*

Cre-	Cre+
49.65	24.25
50.67	34.9
48.29	34.59
	23.6

* ALP+ periosteal thickness was measured and quantified manually using ImageJ software. ALP+ periosteal thickness are presented as averages of four sites (1500 µm from fracture site) in each sample, in 3-4 samples total.

Fig. 4E
PDGFR Deletion

Cre-	Cre+
41.5	19.3
88	17.7
65	27.4

Fig. 5A
Proliferating Cells (no treatment)

AdGFP	AdCre
5.05	4.35
4.85	4.39
4.72	4.59

Proliferating Cells (+PDGF-BB)

AdGFP	AdCre
11.6	10.1
11.3	10.4
11.4	10.1

Fig. 6A
CD140b+

Cre-	Cre+
0.45	0.23
2.69	0.21
1.25	0.25
1.46	0.23

Edu+

Cre-	Cre+
11.2	8.39
13.9	6.22
12.55	3.65

Fig. 6B

CD140b+

Cre-	Cre+
0.85	0.47
0.45	0.52
0.42	0.45
0.57	0.48

EdU+

Cre-	Cre+
19.7	12.3
14.3	12.4
23.8	11.3
